# Wave intensity of aortic root pressure as diagnostic marker of left ventricular systolic dysfunction

**DOI:** 10.1371/journal.pone.0179938

**Published:** 2017-06-19

**Authors:** Bernhard Hametner, Stephanie Parragh, Thomas Weber, Siegfried Wassertheurer

**Affiliations:** 1 Biomedical Systems, Center for Health & Bioresources, AIT Austrian Institute of Technology GmbH, Vienna, Austria; 2 Institute for Analysis and Scientific Computing, Vienna University of Technology, Vienna, Austria; 3 Cardiology Department, Klinikum Wels-Grieskirchen, Wels, Austria; Scuola Superiore Sant'Anna, ITALY

## Abstract

**Background:**

Systolic left ventricular function strongly influences the blood pressure waveform. Therefore, pressure-derived parameters might potentially be used as non-invasive, diagnostic markers of left ventricular impairment. The aim of this study was to investigate the performance of pressure-based parameters in combination with electrocardiography (ECG) for the detection of left ventricular systolic dysfunction defined as severely reduced ejection fraction (EF).

**Methods and results:**

Two populations, each comprising patients with reduced EF and pressure-matched controls, were included for the main analysis (51/102 patients) and model testing (44/88 patients). Central pressure was derived from radial readings and used to compute blood flow. Subsequently, pulse wave analysis and wave intensity analysis were performed and the ratio of the two peaks of forward intensity (SDR) was calculated as a novel index of ventricular function. SDR was significantly decreased in the reduced EF group (2.5 vs. 4.4, P<0.001), as was central pulse pressure, augmentation index and ejection duration (ED), while the QRS-duration was prolonged. SDR and ED were independent predictors of ventricular impairment and when combined with QRS in a simple decision tree, a reduced EF could be detected with a sensitivity of 92% and a specificity of 80%. The independent power of ED, SDR and QRS to predict reduced EF was furthermore confirmed in the test population.

**Conclusion:**

The detection or indication of reduced ejection fraction from pressure-derived parameters seems feasible. These parameters could help to improve the quality of cardiovascular risk stratification or might be used in screening strategies in the general population.

## Introduction

Heart failure represents a serious global health problem that is becoming more and more frequent in the aging population: prognoses in the US predict prevalence to rise by 46% from 2012 to 2030 [1]. Asymptomatic systolic or diastolic dysfunction is common in the population, and is associated with a high risk for hospitalization and mortality [2]. In patients with asymptomatic left ventricular systolic dysfunction, pharmacological treatment may delay or even halt the progression of the disease [2] and the early detection of a potentially impaired systolic function, ideally at a primary care level, may therefore be of great value.

In clinical practice, ventricular function is commonly assessed by echocardiography. Also, electrocardiography (ECG) is recommended as first line investigation for suspected heart failure [2,3]. However, parameters describing pulsatile hemodynamics might be used for diagnosis as well, since an impairment of systolic ventricular function strongly influences the resulting pressure and flow waveforms.

For the noninvasive assessment of aortic pressure, different devices have been validated that provide central pressure from peripheral readings by the means of generalized transfer functions [4,5]. Aortic blood flow can be measured noninvasively using Doppler ultrasound or alternatively estimated from the pressure waveform to simplify the procedure [6].

In this context, an approach based on pressure and flow known as wave intensity analysis (WIA) seems especially promising [7,8]. In WIA, blood pressure and flow velocity measured at the same arterial site are considered and a separation into forward and backward travelling waves can be achieved. Characteristics of wave intensity have been shown to be related to left ventricular ejection and relaxation dynamics [7,8].

Of particular relevance for heart failure, the shape of pressure and flow in the systemic arteries results not only from the mechanical properties of the arteries but from a complex interaction of the ejecting heart and the vasculature. In patients with systolic dysfunction, indices of wave reflections are generally lower than in controls [9–12], indicating a pseudo-healthy status, and the relation of common risk indicators like pulse pressure to outcome was found to be reversed [13], or u-shaped [14]. However, it has not yet been evaluated if these differences can be used as diagnostic markers.

Therefore, the aim of this study was to investigate the performance of measures of pulsatile hemodynamics, derived by pulse wave analysis (PWA) and WIA from non-invasive pressure readings, alone and in combination with ECG-measurements for the discrimination between normal and impaired left ventricular systolic function.

## Methods

### Study population

51 patients with reduced ejection fraction (EF<45%) and 102 controls with normal EF (EF>50%, including 13 patients with heart failure with preserved ejection fraction, HFpEF) matched for gender, age, body mass index and brachial blood pressure were collected at the university teaching hospital Wels-Grieskirchen, Austria. These 153 patients formed the main study population used for primary analysis and the development of classification models. In a second step, a test data set comprising 44 patients with reduced EF and 88 matched controls (including 15 patients with HFpEF) was assembled in the same manner. Patient recruitment for both populations lasted from January 2005 to December 2012. Exclusion criteria for all subjects were arrhythmias (mainly atrial fibrillation), unstable clinical conditions and valvular heart disease exceeding mild severity. In order to have two distinct groups in this pilot study, patients with intermediate EF (45%-50%) were not included. The study was performed within the framework of ongoing studies investigating the role of pulsatile hemodynamics in cardiology [9,15], which were approved by the ethics committee of Upper Austria and written informed consent was obtained from all participants.

### Measurements

Radial pressure waveforms were recorded by applanation tonometry (Millar SPT 301, Millar, Inc., Houston, Texas), calibrated with oscillometric brachial pressure (Omron M5-I, Omron Healthcare, Kyoto, Japan), and processed with the SphygmoCor system (AtCor Medical Pty. Ltd, West Ryde, Australia) to obtain aortic pressure waveforms by applying a generalized transfer function [5]. The estimate of ejection duration (ED) provided by the SphygmoCor system was indexed to heart rate (HR) to obtain the gender-specific ejection time index LVETI [16]. Echocardiographic examination was carried out immediately before or after pressure measurement using a Philips iE33 Ultrasound machine (Philips Medical Systems, Andover, Massachusetts). Left ventricular systolic function was evaluated according to published recommendations [17] and blood flow in the left ventricular outflow tract was acquired by Doppler ultrasound in the apical five chamber view. The duration of cardiac depolarization (QRS-interval) was automatically derived from 12-lead ECGs with the MAC 5500 (GE Healthcare, Little Chalfont, UK) and its inbuilt analysis software and manually checked for reliability.

### Blood flow model and wave intensity analysis

**Fig 1:**
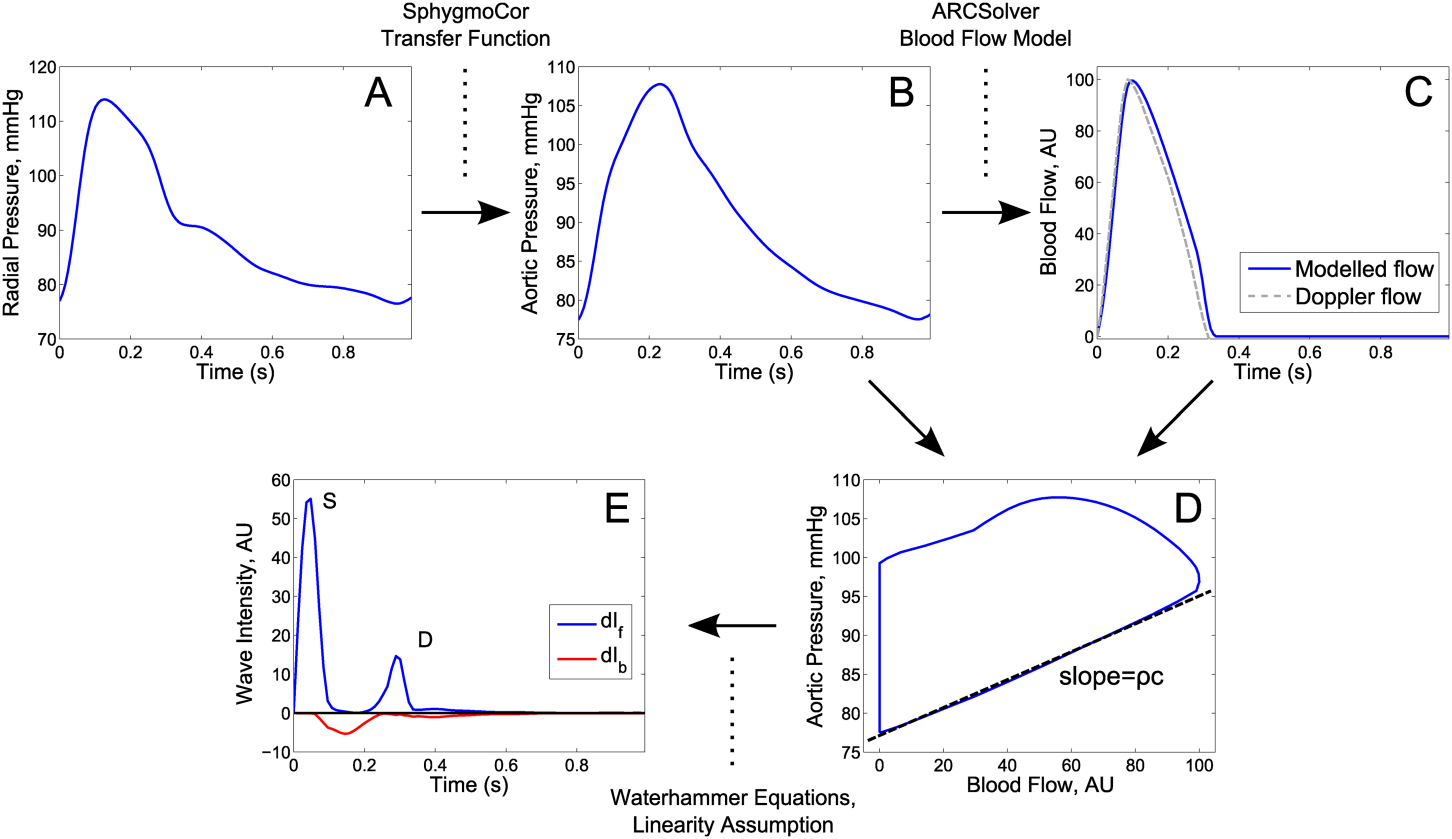
Computational steps applied for wave intensity analysis. From tonometrically measured radial pressure (A), aortic pressure (B) is obtained by a generalized transfer function (SphygmoCor). Aortic blood flow (C) is modelled from aortic pressure (ARCSolver) (or measured by Doppler ultrasound for model validation only). Pulse wave velocity c times blood density ρ is estimated from the slope of the approximately linear part of the PU-loop during early systole (D) to derive forward and backward wave intensity (E).

All computational steps are exemplarily depicted in Fig 1. Aortic pressure P was used to obtain aortic blood flow Q with the ARCSolver algorithms (AIT Austrian Institute of Technology GmbH, Vienna, Austria), which are based on a Windkessel model to describe the dynamic relation between pressure and flow plus a minimal work criterion [6]. Q scaled between a minimum of 0 arbitrary units to a maximum of 100 arbitrary units was used as an estimate of the shape of flow velocity U. For WIA, the changes dP and dU per time step were computed and separated into forward (subscript f) and backward (subscript b) travelling components using the Waterhammer equations dP_f,b_ = ±ρc dU_f,b_ and a linearity assumption: dP = dP_f_+dP_b_, dU = dU_f_+dU_b_ [7]. ρ thereby denotes the blood density (1050 kg/m^3^) and c the wave speed which was estimated with the PU-loop method [18]. The forward and backward wave intensities are then given by dI_f,b_ = dP_f,b_*dU_f,b_. dI_f_ generally shows two peaks, denoted as S and D, which are supposed to hold information on systolic and late-systolic/early-diastolic ventricular function [8,19]. Since wave intensity is based on differences of pressure and flow, the S-peak is related to the maximum derivative of left ventricular pressure, while the D-peak is related to the time constant of the pressure decay. The S to D ratio (SDR) is intended as a novel, relative index of systolic function, combining early- and late-systolic pressure and flow dynamics. SDR is independent of the absolute scaling of flow velocity, as this cancels out due to the quotient of WIA parameters. All computations were carried out using Matlab R2011b (The Mathworks, Inc, Natick, Massachusetts).

### Evaluation of the diagnostic value

The diagnostic value of the various parameters was analyzed in four stages. First, all parameters were evaluated separately in univariable analyses and their performance was compared. In the second stage, combinations of pressure-derived parameters were analyzed and statistical models based on logistic regression analysis were used to identify the strongest independent predictors of a reduced EF. In the third stage, stepwise classification schemes based on the results of the two previous stages were formulated and investigated. These schemes represent easily applicable decision trees that, in contrast to the results of classic statistical models, could be directly transferred to clinical practice. Finally, the proposed scheme was tested in the second data set using the thresholds obtained in stage three.

### Statistics

For the statistical evaluation, Medcalc Statistical Software version 15.6.1 (MedCalc Software bvba, Ostend, Belgium) and Matlab R2011b were used. Continuous data are presented as mean (standard deviation) or median [inter-quartile range], according to the results of a Kolmogorov-Smirnov test for normality, categorical data as total number (percentage). Between-group differences of continuous data were quantified with an unpaired t-test (equal variances) or Welch test (unequal variances) if normally or log-normally distributed and with the Mann-Whitney-U test elsewise. For proportions, the chi squared test was used. The performance of the different parameters as binary classifiers of a reduced EF was analyzed using logistic regression, receiver-operator characteristics (ROC) and the area under the ROC curve (AUC). AUCs were compared using the method of DeLong as implemented in MedCalc. The added predictive value of single variables in the stepwise classification schemes was evaluated by the net reclassification improvement as described in [20]. Statistical significance was assumed at a 5% level.

## Results

Presented results refer to the main (development) population only, unless explicitly indicated as pertaining to the test population.

The main study population consisted predominantly of male patients (93%) and mean age was 59.9 years (range 27 to 87) for patients with reduced EF and 59.7 years (range 33 to 80) for controls. Anthropometric measures (height, weight, body mass index) as well as the prevalence of coronary artery disease, hypertension and diabetes were comparable between the groups, but antihypertensive medication was more common in reduced EF patients. See Table 1 for a detailed summary of the baseline characteristics for both the development and the test population.

**Table 1:** Baseline characteristics of both patient groups in the main and the test population.

	Main population		Test population	
	Severely reduced EF	Normal EF	Severely reduced EF	Normal EF
Patients	51	102	44	88
Gender (male/female)	47/4	96/6	39/5	78/10
Age, years	59.9 (12.3 SD)	59.8 (10.8 SD)	60.5 (10.7 SD)	60.1 (10.4 SD)
Height, cm	174 (7.15 SD)	175 (7.8 SD)	173 (7.08 SD)	173 (8.76 SD)
Weight, kg	86.9 (16.4 SD)	86.9 (13.8 SD)	85.7 (16.3 SD)	86.1 (18.3 SD)
Body mass index, kg/m^2	28.7 (4.49 SD)	28.6 (4.27 SD)	28.6 (5.35 SD)	28.7 (5.5 SD)
Body surface area, m^2	2.01 (0.206 SD)	2.02 (0.172 SD)	2.00 (0.188 SD)	2.00 (0.228 SD)
Ejection fraction, %	27.8 (8.10 SD) *	69.7 (7.56 SD) *	28.3 (7.31 SD) *	68.2 (9.07 SD) *
Hypertension	30 (59%)	66 (65%)	25 (57%)	61 (69%)
Diabetes	14 (27%)	17 (17%)	10 (23%)	13 (15%)
Coronary artery disease	27 (53%)	45 (44%)	17 (39%)	41 (47%)
ACE/ARB, n(%)	43 (84%) *	35 (34%) *	39 (89%) *	47 (53%) *
Beta blocker, n (%)	42 (82%) *	51 (50%) *	34 (77%) *	38 (43%) *
Calcium channel blocker, n (%)	5 (10%)	9 (9%)	5 (11%)	12 (14%)
Diuretic, n (%)	30 (59%) *	24 (24%) *	30 (68%) *	18 (20%) *
NO donator, n (%)	5 (10%)	11 (11%)	7 (16%)	11 (13%)
Acetylsalicylic acid, n (%)	35 (69%)	83 (81%)	35 (80%)	68 (77%)
Statin, n (%)	17 (33%)	42 (41%)	14 (32%)	40 (45%)

EF, ejection fraction; ACE, angiotensin converting enzyme inhibitors; ARB, angiotensin receptor blocker. Hypertension was defined as office BP > 140/90 or preexisting treatment for hypertension. Values are given as mean (standard deviation) or number (percentage).

* indicates a significant difference in group-wise comparison (severely reduced vs. normal ejection fraction); P-values were <0.001 for all detected differences.

Table 2 depicts the results of PWA, WIA and ECG for both patient groups in the main population. HR and QRS-duration were increased, whereas ED and ED indexed to HR (LVETI) were reduced in the low EF group compared to controls. Although brachial pressures were well matched, estimated central pulse pressure (PP) showed statistically significant differences between the groups and augmentation index was lower for reduced EF. Also, the S to D ratio derived by WIA was significantly lower in the reduced EF group (SDR: 2.5 [1.0, 3.6] vs. 4.4 [3.4, 5.9], P<0.001). This reduction was comparable to the one obtained using Doppler flow measurements (SDR Doppler: 2.5 [1.7,3.3] vs. 5.1 [4.0,6.6], P<0.001) and the difference between SDR and SDR Doppler was non-significant across the study population.

**Table 2:** Pulse wave analysis, wave intensity analysis and ECG parameters.

	Severely reduced EF	Normal EF	P-value
**Pressure-derived parameters**			
Heart rate, bpm	74.8 (13.4 SD)	64.3 (10.3 SD)	<0.001
Ejection duration, ms	268 (27.6 SD)	309 (26.0 SD)	<0.001
LVETI, ms	394 (19.8 SD)	417 (18.5 SD)	<0.001
brachial SBP, mmHg	123 (20.8 SD)	127 (13.3 SD)	0.30
central SBP, mmHg	111 (18.7 SD)	117 (12.2 SD)	0.06
brachial DBP. mmHg	78.0 (13.8 SD)	79.0 (9.13 SD)	0.63
central DBP, mmHg	78.7 (14.1 SD)	79.8 (9.18 SD)	0.62
brachial PP, mmHg	45.5 (14.1 SD)	47.8 (11.7 SD)	0.28
central PP, mmHg	32.3 (12.2 SD)	36.7 (9.89 SD)	0.02
AIx, -	0.165 (0.086 SD)	0.247 (0.108 SD)	<0.001
SDR, -	2.5 [1.98,3.61]	4.41 [3.4,5.93]	<0.001
**Doppler Ultrasound**			
Doppler SDR, -	2.49 [1.71,3.31]	5.05 [4.03,6.56]	<0.001
**ECG**			
QRS-interval, ms	110 [97,133]	92 [86,100]	<0.001

Results are presented as mean (standard deviation) and median [inter-quartile range] for normally and non-normally distributed data respectively. P-values present the results of group-wise comparisons (severely reduced vs. normal ejection fraction). LVETI, left ventricular ejection time index; SBP, systolic blood pressure; DBP, diastolic blood pressure; PP, pulse pressure; AIx, augmentation index; SDR, ratio of the first (S) to second (D) peak of forward wave intensity.

**Fig 2:**
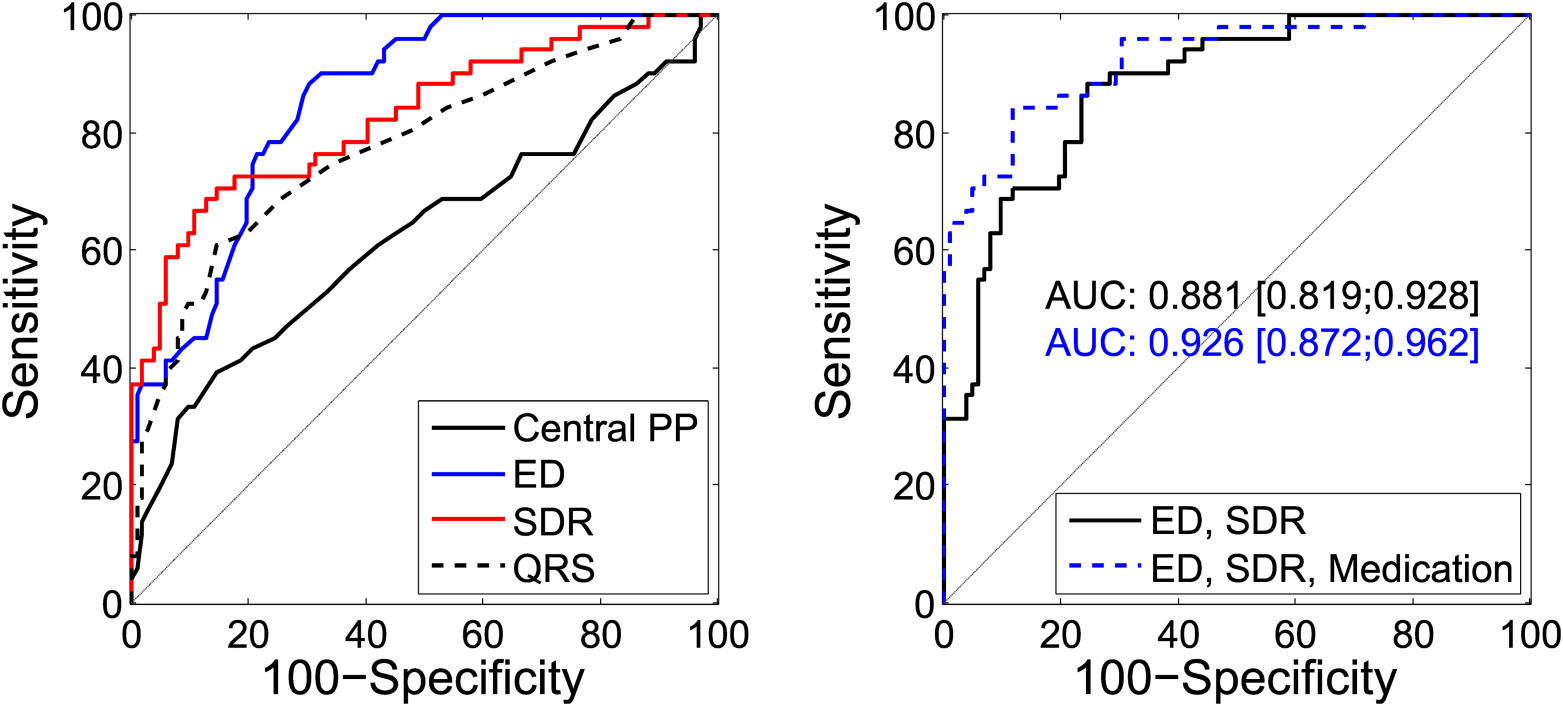
ROC curve analysis. Left: Comparison of the ROC curves obtained with central pulse pressure (PP), ejection duration (ED), S to D ratio (SDR) and QRS-duration. Right: ROC curves obtained with a combination of ED and SDR, and ED and SDR when adjusted for medication. Area under the curve (AUC) and 95% confidence interval are given in the same colors.

The performance of the different parameters as binary classifiers of a systolic dysfunction are quantitatively presented in Table 3 and graphically depicted in Fig 2. Blood pressure levels showed the worst performance, whereas ED, LVETI and SDR all reached an AUC of above 0.8. The AUC obtained with SDR was smaller than its counterpart obtained with the flow velocity acquired by Doppler ultrasound (0.83 to 0.88). However, a pairwise comparison showed no evidence of a statistical difference (difference AUC: 0.05, 95% confidence interval of [-0.32,0.14], P = 0.22). Using Doppler flow, the S and D peak values could also be evaluated individually. Here the AUCs were significantly lower than for the SDR (difference SDR to S: 0.12, 95% confidence interval of [0.05,0.19], P<0.001; difference SDR to D: 0.28, 95% confidence interval of [0.18,0.39], P<0.001).

**Table 3:** Univariable logistic regression models for EF-status (0: Normal, 1: Reduced).

Parameter	OR	95% CI OR	AUC	SE	95% CI AUC	Percent correctly classified	Criterion
Heart rate, per bpm	1.077	[1.043,1.112]	0.736	0.0437	[0.659,0.804]	71.24%	≥70
Ejection duration, per ms	0.948	[0.931,0.965]	0.855	0.0295	[0.789,0.906]	75.82%	≤297
LVETI, per ms	0.940	[0.919,0.961]	0.814	0.0342	[0.744,0.873]	71.90%	≤414
brachial PP, per mmHg	0.985	[0.958,1.013]	0.559	0.0524	[0.476,0.639]	69.93%	≤33
central PP, per mmHg	0.961	[0.929,0.994]	0.624	0.0513	[0.543,0.701]	69.93%	≤26
AIx, per 0.01	0.923	[0.889,0.959]	0.744	0.0407	[0.667,0.811]	66.01%	≤0.24
SDR, per 1	0.385	[0.268,0.551]	0.829	0.0371	[0.760,0.885]	81.70%	≤2.88
QRS-interval, per ms	1.060	[1.036,1.085]	0.778	0.0412	[0.703,0.841]	77.12%	≥104
S peak Doppler, per 1	0.970	[0.957, 0.984]	0.760	0.0428	[0.684, 0.825]	77.78%	≤46.1
D peak Doppler, per 1	1.025	[0.992, 1.058]	0.594	0.0488	[0.512, 0.673]	55,56%	≥16.5
SDR Doppler, per 1	0.341	[0.239, 0.487]	0.879	0.0315	[0,816, 0.926]	81,05%	≤3.87

Percent correctly classified and the corresponding criterion refers to the point with maximum Youden Index J = Sensitivity+Specificity-1. OR, odds ratio; CI, confidence interval; AUC, area under receiver operating characteristics curve; SE, standard error; abbreviations as in Table 2, additionally S (or D) peak Doppler: S (or D) peak value of wave intensity calculated with pressure and Doppler flow waves.

Stepwise logistic regression analysis was applied to all pressure-derived parameters to identify the best predictors. The final model consisted of ED and SDR and reached a combined AUC of 0.88 [0.82, 0.93], while HR, LVETI, augmentation index as well as central and brachial PP were not included. With this model, the Youden index was maximal at a sensitivity of 76% and a specificity of 88% resulting in 80% correctly classified subjects. Adjustment for medication as a confounding factor increased the AUC further to 0.93 [0.87, 0.96] and the percentage of correctly classified subjects to 87% with a sensitivity of 88% and a specificity of 84%, compare Fig 2 and Table 4.

**Table 4:** Stepwise multivariable logistic regression models for EF-status (0: Normal, 1: Reduced) using pressure-derived parameters with and without adjustment for medication.

Parameter		95% CI
**Model including pressure-derived parameters**		
ED, OR per ms	0.957	[0.939, 0.975]
SDR, OR per 1	0.533	[0.364, 0.780]
Not included	HR, LVETI, central PP, brachial PP, AIx	
AUC	0.881	[0.819, 0.928]
**Model including pressure-derived parameters and medication**		
ED, OR per ms	0.956	[0.935, 0.977]
SDR, OR per 1	0.608	[0.404, 0.915]
Beta blocker, yes = 1	4.190	[1.306, 13.440]
ACE/ARB, yes = 1	6.133	[1.861, 20.215]
ASA, yes = 1	0.246	[0.069,0.881]
Not included	HR, LVETI, central PP, brachial PP, AIx, CCB, diuretic, NO-donator, statin	
AUC	0.926	[0.872, 0.962]

A variable was included if P<0.05 and removed if P>0.1. OR, odds ratio; AUC, area under the ROC curve; ED, ejection duration; SDR, S to D ratio; HR, heart rate; LVETI, left ventricular ejection time index; AIx, augmentation index; ACE, angiotensin-converting enzyme inhibitors, ARB: angiotensin II receptor blocker; ASA, acetylsalicylic acid; CCB, calcium channel blocker;

Table 5 shows the results of a stepwise classification scheme using first SDR, then ED and finally QRS to determine or rule-out a reduced EF. The corresponding criteria were chosen such that the false positive rate (100-Specificity) and false negative rate (100-Sensitivity) were ≤5%. In the first step, patients with SDR≤2.5 were classified as “YES”, having reduced EF, those with SDR>5.6 as “NO”, having normal EF, leaving 91 patients unclassified. These were further analyzed using ED (“YES” for ED≤257 ms, “NO” for ED>310 ms), resulting in a total net reclassification improvement of 43%, which was mainly due to an improved classification of patients with normal EF. The remaining 45 subjects were finally classified with QRS (“YES” for QRS≥126 ms, “NO” for QRS<84 ms), significantly improving the classification further by 23%. This improvement was again stronger for the normal EF group. Overall, 132 out of 153 could be classified, thereof 119 correctly. More specifically, 73% of the patients with reduced EF were correctly assigned to “YES”, and 80% of the controls to “NO”, while 20% of the reduced EF and 11% of the normal EF group remained unclassified. Since the order of the variables may influence the results, a second scheme is given in Table 5 for comparison, which is again based on SDR, ED and QRS, yet starting with QRS. Also in this case, each step significantly improved the overall classification.

**Table 5:** Approach to diagnose or exclude a reduced ejection fraction by a stepwise classification scheme.

	Criteria		Categories				
	YES	NO	YES (rEF/nEF)	Inconclusive (rEF/nEF)	NO (rEF/nEF)	NRI total/rEF/nEF	P-value total/rEF/nEF
Model 1							
SDR	≤2.5	>5.6	31 (26/5)	91 (23/68)	31 (2/29)		
ED	≤257	>310	41 (33/8)	45 (15/30)	67 (3/64)	0.43/0.12/0.31	<0.001/0.03/<0.001
QRS	≥138	<98	46 (37/9)	21 (10/11)	86 (4/82)	0.23/0.06/0.17	<0.001/0.18/<0.001
Model 2							
QRS	≥124	<84	23 (18/5)	111 (31/80)	19 (2/17)		
SDR	≤2.5	>5.6	42 (33/9)	67 (15/52)	44 (3/41)	0.47/0.27/0.20	<0.001/<0.001/<0.001
ED	≤254	>299	50 (39/11)	24 (8/16)	79 (4/75)	0.41/0.10/0.31	<0.001/0.06/<0.001

For this approach, subjects were classified as having severely reduced EF (YES) or not (NO) according to the criteria given in the first two columns, starting with the parameter in the first row. If none of the criteria applied, results were inconclusive and the parameter in the next row was used for further classification. Criteria were optimized with regards to the false positive and false negative rates (max 5%). NRI indicates the improvement from one step to the next and is given for the total population as well as for the reduced and normal EF group separately. NRI, net reclassification improvement; rEF, reduced ejection fraction; nEF, normal ejection fraction; ED, ejection duration; SDR, S to D ratio.

Baseline characteristics of the test population were very similar to the main, development population, see Table 1, and pressure-derived parameters as well as QRS-duration were in the same range, compare Table 6. However, differences in HR were less pronounced between the groups and even though central PP was again lower in the reduced EF group, this reduction did not reach statistical significance.

**Table 6:** Pulse wave analysis, wave intensity analysis and ECG parameters in the test population.

	Severely reduced EF	Normal EF	P-value
**Pressure-derived parameters**			
Heart rate, bpm	72.1 (12.3 SD)	66.4 (12.3 SD)	0.01
Ejection duration, ms	266 (27.0 SD)	305 (27.8 SD)	<0.001
LVETI, ms	388 (24.1 SD)	417 (18.6 SD)	<0.001
brachial SBP, mmHg	124 (19.3 SD)	125 (15.7 SD)	0.75
central SBP, mmHg	111 (18.1 SD)	114 (15.3 SD)	0.37
brachial DBP. mmHg	78.3 (12.8 SD)	77.9 (11.9 SD)	0.86
central DBP, mmHg	78.9 (12.9 SD)	78.8 (12.1 SD)	0.99
brachial PP, mmHg	45.4 (12.1 SD)	46.8 (9.79 SD)	0.48
central PP, mmHg	32.4 (10.5 SD)	35.1 (8.57 SD)	0.11
AIx, -	0.190 (0.121 SD)	0.221 (0.105 SD)	0.13
SDR, -	2.61 [1.88,3.28]	4.75 [3.45,6.37]	<0.001
**ECG**			
QRS-interval, ms	120 [98,146]	91 [86,102]	<0.001

Results are presented as mean (standard deviation) and median [inter-quartile range] for normally and non-normally distributed data respectively. P-values present the results of group-wise comparisons (severely reduced vs. normal ejection fraction). LVETI, left ventricular ejection time index; SBP, systolic blood pressure; DBP, diastolic blood pressure; PP, pulse pressure; AIx, augmentation index; SDR, S to D ratio.

Results obtained with the classification scheme from Table 5 (model 1) using prescribed cutoff-values are finally presented in Table 7. Again, each step resulted in a significant improvement and 70% of the patients with reduced EF and 76% of the normal EF group were classified correctly, while 11% and 13% of the two groups remained unclassified.

**Table 7:** Approach to diagnose or exclude a reduced ejection fraction by a stepwise classification scheme with fixed criteria in the test population.

	Criteria		Categories				
	YES	NO	YES (rEF/nEF)	Inconclusive (rEF/nEF)	NO (rEF/nEF)	NRI total/rEF/nEF	P-value total/rEF/nEF
SDR	**≤2.5**	**>5.6**	27 (20/7)	72 (22/50)	33 (2/31)		
ED	**≤257**	**>310**	37 (28/9)	38 (11/27)	57 (5/52)	0.33/0.11/0.22	<0.001/0.13/<0.001
QRS	**≥138**	**<98**	41 (31/10)	16 (5/11)	75 (8/67)	0.16/0.00/0.16	0.03/1.00/<0.001

Subjects were classified as having severely reduced EF (YES) or not (NO) according to the criteria obtained in the main population given in Table 5. Abbreviations as in Table 5.

## Discussion

In this study, a new marker of impaired systolic function, the ratio of the first to second peak of forward wave intensity (SDR) derived from pressure waveforms alone, was introduced and investigated as a discriminator between subjects with reduced EF and controls. Furthermore, the combination of SDR with other pressure-derived measures and QRS-duration from ECG was examined.

The influence of left ventricular impairment on the shape of the central pressure wave is reflected in the reduction of central PP and augmentation index found in this study as well as in previous works [9–11]. Also, ED and HR are important determinants of the pulse waveform and were both altered in the reduced EF group, whereby the differences in LVETI imply that ED was reduced beyond the effect of HR elevation. These changes in HR and ED have been reported before [9–11,21] and might be explained by the inability of the impaired ventricle to overcome late systolic load (shorter ED), leading to an increase in heart rate to compensate the resulting lower stroke volume [22]. Of note, HR was higher despite the higher prevalence of beta blocker use in the reduced EF group. PP amplification, the ratio of peripheral to central PP, assessed with the SphygmoCor system has been found to increase linearly with HR [23]. This is in line with our finding that PP amplification tends to be higher in patients with reduced EF compared to controls resulting in a larger difference in central than in brachial PP between the groups. More precisely, due to the matching criteria, peripheral PP did not differ significantly between groups for both populations, while central PP differed with a borderline significance (p = 0.02 for main population, p = 0.11 for test population). Also the augmentation index has been reported to be influenced by temporal effects [24,25] as well as cardiac properties [26].

While augmentation index and PP are both indirect measures of and strongly influenced by wave reflections, forward wave intensity is comprised of forward travelling waves only. These forward waves are generated by the left ventricle and result in two dominant peaks in the wave intensity dI_f_. The first peak S is related to left ventricular contractility [8], whereas the second peak D is associated with the beginning of relaxation [7]. Their ratio could thus be seen as an index that combines the characteristics of the left ventricle during the entire mechanical systole. The lower values of SDR observed in the reduced EF group are in line with these theoretical considerations as well as with results from Sugawara et al. [8] and Curtis et al. [19], who both found a reduction in the first but not the second peak of wave intensity in subjects with systolic heart failure compared to controls. Results from the present study using Doppler flow also indicate that the main effect is a reduction of the S-peak, but the ratio of S and D has a significantly higher AUC and thus an even higher discriminative power than the S-peak alone.

A similar approach was taken by Ntsinjana and coworkers to analyze ventricular function in children with HFpEF [27]. Equivalently to SDR, they calculated the ratio of forward compression wave and forward expansion wave from wave intensity derived with area and velocity obtained from cardiovascular magnetic resonance phase-contrast data. A significant reduction was found for patients with HFpEF compared to controls. Although patients had a normal ejection fraction, the authors speculated that this indicates a load-independent systolic dysfunction. This finding should be interpreted in context with another recent work by Gu and coworkers in patients with diastolic dysfunction [28]. The authors linked systolic to diastolic function via an impaired shortening deactivation and a reduced so-called early ejection fraction, despite preserved total ejection fraction. This early ejection fraction was determined during early systole, thus it is potentially related to the S-peak in WIA. Clearly, further studies are needed to investigate these relations and the role of SDR in heart failure beside patients with HFrEF, who have been in focus in this current study.

As a ratio, SDR is virtually independent of the sampling frequency, making its value comparable between different investigators using different instruments. Furthermore, the computation of SDR is not affected by the absolute scaling of blood flow, which enables the use of a (volumetric) blood flow model for the estimation of flow velocity. The model used here has been investigated previously in patients with and without heart failure with reduced EF [6,24,29]. In both cases, modelled flow showed a good agreement with Doppler flow measurements and the qualitative and quantitative behavior of the derived PWA parameters was comparable. Also in the present work, differences in SDR as well as in the derived AUC between modelled and measured flow were non-significant. The use of a flow model for the computation of SDR greatly facilitates the data acquisition process and represents an important criterion to enable its implementation in day-to-day clinical practice.

In ROC curve analysis, ED and SDR reached the largest AUC from all pressure-derived parameters considered in this study. Furthermore, the results of multivariable logistic regression analysis indicated no independent additive association of either PP, augmentation index, HR or LVETI to EF-status beyond ED and SDR. This might be explained by the intrinsic link between HR and ED on the one hand and the impact of HR and ED on both PP and augmentation index on the other hand [23,25]. In contrast, SDR seems to reflect aspects of ventricular function that are not covered by ED. This is further underlined by the different shapes of their ROC curves, while using SDR and ED together combines the advantages of both, as presented in Fig 2. Overall, the AUC of 0.88 obtained with the combination of ED and SDR implies a satisfactory discrimination between patients with normal and reduced EF, which is further improved if medication is adjusted for. Sensitivity and specificity lie within the range of 76%–92%. Despite high values for the AUC, the sensitivity might not be sufficiently high for a rigorous clinical test, but since the maximum Youden-index is used here as cutoff-criteria, application-specific cutoff-values can enhance the sensitivity if needed.

Systolic heart failure is reflected in various abnormalities in the ECG signal, including temporal and morphological characteristics [3], and a normal ECG makes left ventricular systolic dysfunction (LVSD) very unlikely [3,30]. Assessment of the QRS-duration is explicitly recommended as part of the diagnostic investigation in suspected heart failure by the European Society of Cardiology [3] and it has been investigated before as an identifier of LVSD [31,32]. The observed prolongation in patients with reduced EF is in line with previous findings [31,32] and the specificity of 85% obtained in the present work for QRS≥104 ms coincides with the 84% obtained by Murkofsky et al. [31] for QRS≥100 ms. Sensitivity was even higher (61% compared to 44%).

Based on a community-based study performed in Germany, Fischer et al. concluded that echocardiographic screening for LVSD cannot be recommended in the unselected, middle-aged population because asymptomatic LVSD is rare and most patients with LVSD present with cardiovascular comorbidities [33]. However, even in patients with suspected heart failure, left ventricular function is often not assessed by primary care physicians, leading to possible misdiagnoses [34]. Therefore, non-invasive methods for either preselection or for indicating further examination could help to improve the early diagnosis and treatment decision in a primary care setting. This idea has been investigated before with regards to ECG, yet using ECG alone was found to be of limited clinical use [30]. The decision tree given in Table 7 (model 1) represents a theoretical pre-echocardiographic screening test based on a combination of pressure-derived parameters and ECG. A similar approach has been presented by Weber et al. for the diagnosis of heart failure with preserved EF [35]. The results obtained with the second model, i.e. with a changed order of the variables, confirmed the independency between the measures: improvement was still significant for each parameter included and the cut-off values remained mostly unchanged. The association of the chosen parameters to an impairment of ventricular systolic function was further corroborated by the classification results achieved in the test population, indicating that the observed relations are not restricted to the initial population used for model development.

Recently, operator-independent methods based on oscillometric pressure cuffs have been introduced for the assessment of central hemodynamics [4]. Thus, all parameters used in this work could potentially be obtained automatically using a pressure cuff and an ECG, making them easily assessable also at a primary care level.

### Limitations

The study population was collected in a 1:2 fashion regarding the proportion of patients with reduced EF to controls, which does not reflect the real, low prevalence of approximately 1% for systolic heart failure and 2% for LVSD in general population [3,33]. However, the prevalence of systolic heart failure increases with age, reaching more than 5% in age groups above 70 [3]. The control groups included a number of patients with HFpEF, but due to the limited amount of patients with HFpEF in the study fulfilling the matching criteria, two control groups solely based on HFpEF patients could not be set up. This would allow distinguishing whether controls with and without HFpEF will be identified correctly in a comparable amount. The fact that predominantly male subjects (91%) were included in this study has to be stated as another limitation. However, also previous studies in systolic heart failure show an unbalanced gender distribution [10,11] (between 80% and 97% male), which might be caused by the unequal prevalence of LVSD with regards to gender, which was found to be about 2.5 times higher in men than in women [36]. The applicability of the proposed scheme in the general population has to be investigated in future studies and bigger data sets are necessary to derive optimal threshold values. In the current work, the false negative rate was rather high (~18%) in the test population, but higher sensitivity could be achieved by adopting the cutoff values. Moreover, sensitivity and specificity were rated equally important in this study, but could be chosen differently for clinical application.

### Conclusion

The detection or indication of reduced ejection fraction from parameters derived from pulse wave readings seems feasible. These parameters could help to improve the quality of cardiovascular risk stratification and might potentially be incorporated into cheap, noninvasive screening strategies in the general population.
